# Shared social identity and media transmission of trauma

**DOI:** 10.1038/s41598-023-33898-2

**Published:** 2023-07-18

**Authors:** Daniel P. Relihan, Nickolas M. Jones, E. Alison Holman, Roxane Cohen Silver

**Affiliations:** 1 Department of Psychological Science, 4201 Social and Behavioral Sciences Gateway, University of California, Irvine, CA 92697-7085 USA; 2 Sue & Bill Gross School of Nursing, Room 4517, Nursing and Health Sciences Hall, University of California, Irvine, CA 92697 USA; 3 Department of Medicine and Program in Public Health, University of California, Irvine, CA 92697 USA

**Keywords:** Psychology, Human behaviour

## Abstract

When an individual or group trauma becomes a shared public experience through widespread media coverage (e.g., mass violence, being publicly outed), sharing a social identity with a targeted individual or group of victims may amplify feelings of personal vulnerability. This heightened perceived threat may draw people to engage with trauma-related media because of increased vigilance for self-relevant threats, which can, in turn, amplify distress. We studied this possibility among two U.S. national samples following the 2016 Pulse nightclub massacre in Orlando, FL (N = 4675) and the 2018 Dr. Christine Blasey Ford and Judge Brett Kavanaugh Supreme Court Senate hearings (N = 4894). Participants who shared LGBT or Hispanic identities with Pulse massacre victims reported greater exposure to massacre-related media and acute stress. Participants who shared Dr. Blasey Ford’s identities as a victim of interpersonal violence and a Democrat reported more hearings-related media exposure and acute stress. Indirect effects of shared single identity on acute stress through self-reported event-related media exposure emerged in both studies. Results for sharing dual identities with victims were mixed. These findings have implications for media use and public health.

## Introduction

Traditional and social media frequently disseminate news of group-based traumas (e.g., terrorist attacks, mass shootings) widely. With increasing frequency, graphic detail, and instantaneous reporting, the negative consequences of exposure to media coverage of these events are demonstrated in two decades of correlational and prospective research^1–11^. For example, exposure to media coverage of trauma was associated with acute stress, posttraumatic stress, and depression following the September 11, 2001, terrorist attacks^4–8^, 1995 Oklahoma City bombing^9^, 2002 Washington D.C. sniper shootings^10^, and 2013 Boston Marathon bombings^11^. Thus, media coverage of traumatic events may transmit distress to entire populations beyond those locally affected by it. Considering the breadth and depth of exposure to these events provided by modern media, it is important to understand who might be vulnerable to such media exposure and its negative psychological effects.

Collective stressors frequently occur among homogeneous social groups. For instance, victims of the 2017 Route 91 concert mass shooting in Las Vegas, Nevada were predominantly country music fans, the 2018 Pittsburgh, Pennsylvania synagogue shooting victims were Jewish, and the 2019 Christchurch massacre victims in New Zealand were Muslim. As the trauma is broadcast to people around the world, some may identify with the targeted social groups, placing them at increased risk for distress. Personal identities are the unique aspects of the self that individuate a person from others^12,13^. Social (collective) identities are depersonalized perceptions of the self in terms of social groups, where the self and social ingroup members become interchangeable^13^. People often come to identify with a group by incorporating the group’s identity into their self-concept and attaching emotional significance to that group’s membership^14,15^. When made contextually salient, social identities can be used as a lens through which to interpret information, guide behavior, and experience emotion^14–21^. Hence, an attack on one’s ingroup may be perceived as an attack on oneself, leading to an identity-congruent response such as feelings of anxiety and threat of vulnerability to a similar experience. Indeed, prior research suggests that individuals who share similarities with trauma victims report more depression and distress^22–26^.

However, social identities are more than just perceived similarities. Social identities extend one’s sense of self to ingroup members such that consequences for an individual group member are psychologically and emotionally tied to consequences for other ingroup members^27,28^. Moreover, social identities can be based on shared experiences rather than physical features, and this is especially true for more intense shared experiences. Sharing similar experiences in war, for example, helps mold soldiers’ bonds into the social identity of veterans^27,28^. Two veterans need not share physical attributes, nor even have been in the same war, to share the emotional and psychological bond that comes with being a veteran. They are treated by people the same way, such as with particular deference, and this helps further consolidate their identification with the group. They also have access to the same social identity-specific resources, like veterans' benefits and organizations. Furthermore, being a veteran can be both a personal identity (i.e., it is what is unique about an individual across most social environments) and a social/collective identity (i.e., it is what emotionally and psychologically connects the individual to ingroup members in a way that blurs perceptions of the self and ingroup others). Given that social groups function, in part, to promote identification among members^29^ and can lead to experiencing group-congruent emotions^20,21^, those who share social identities with victims of traumatic experiences that are broadcast widely may share their feelings of threat, anxiety, and distress.

A growing body of research over the past decade suggests that social identities shape people’s physiological and psychological responses to threat^27–36^. The social identity approach to clinical and health psychology recognizes that people do not just experience and express attitudes, emotions, and behaviors as individuals in a physical environment, but also as group members in a social environment^27,28,35,36^. However, this work has largely focused on the role of single shared social identities in direct experiences of trauma, leaving unanswered whether sharing *multiple* social identities with people who have experienced a publicly broadcasted trauma (e.g., mass shooting, being outed) is associated with greater distress following that event.

Moreover, when a person or group’s trauma is shared widely through the media, individuals who share identities with the trauma victim(s) may also be at risk of distress indirectly from increased exposure to event-related media coverage. Individuals who feel threatened or anxious have shown an attentional bias toward threatening stimuli^37,38^, and threatening visual stimuli activate fear and capture attention^39^, especially when relevant to the self^40,41^. Therefore, media coverage of trauma—whether a personal or collective experience—may be especially attention-grabbing for people who share the social identities of the victims. Consequently, victims’ ingroup members may turn to event-related media coverage for information to mitigate feelings of anxiety, ambiguity, and uncertainty^42^. Yet, exposure to this media coverage may have the opposite effect by *increasing*, rather than decreasing, distress^1–11,43^. The present research adds to the social, political, and health psychology literature by examining—among two large U.S. national samples—the roles of shared social identities in media exposure and distress following two public events in the U.S. where the trauma was widely disseminated by the media.

## 2016 Orlando Pulse nightclub massacre

On June 12, 2016, a lone gunman entered Pulse nightclub in Orlando, Florida, and opened fire, injuring 53 and killing 49 people. At the time, the attack was the largest mass shooting in U.S. history. However, it was a unique event in another respect: the victims were predominantly lesbian, gay, bisexual, or transgender (LGBT) and Hispanic. On the evening of the attack, Pulse, an LGBT-oriented nightclub, hosted a Latin-themed night as part of LGBT Pride month celebrations. As a result, approximately 90% of the victims were both LGBT and Hispanic^44^.

Both LGBT and Hispanic individuals tend to be disadvantaged in the U.S., and social identities are not experienced independently of each other. A multiple-identities approach states that the negative impact of belonging to more than one disadvantaged social identity has multiplicative repercussions for negative psychological experiences^45,46^. Research that focuses on a single identity may not fully capture experiences produced from belonging to multiple social groups. For instance, individuals who identify as both LGBT and Hispanic may experience compounded effects of racism in their LGBT communities and heterosexism in their Hispanic communities. We investigated the roles of LGBT and Hispanic identities in Americans’ reactions to the Pulse nightclub attack, and how the interaction of these two identities might be related to greater negative psychological responses. We hypothesized that:

1. Participants who share the salient identities of the Pulse nightclub massacre victims (i.e., LGBT [*n* = 287] or Hispanic [*n* = 478]), would report greater exposure to media coverage about the massacre and greater acute stress than non-LGBT and non-Hispanic participants, respectively,
2. Participants who identify as both LGBT and Hispanic (*n* = 50) would report significantly greater Pulse massacre-related media exposure and greater acute stress than those who identify as one or neither identity, and
3. There would be indirect effects of LGBT and Hispanic identities, and their interaction, on acute stress through self-reported Pulse nightclub massacre media exposure.

Participants consisted of *N* = 4675 panelists from GfK KnowledgePanel, an independent survey research company that uses addressed-based sampling to randomly sample and recruit participants across the U.S. Panelists received free internet or, if already web-enabled, compensation for their participation. Data collection began five days after the Pulse nightclub massacre on Friday June 17, 2016, and lasted until Friday July 22, 2016. Participants reported the average daily number of hours they watched or listened to media coverage about the shooting in the days following the massacre for five types of media sources, including television, radio, online news sources, pictures and/or videos on social media, and news or text updates on social media. Participants also reported their distress about the massacre on a standardized multi-item measure of acute stress^47^. Age, gender, education, employment status, marital status, prior mental health diagnoses, and direct exposure to the Pulse nightclub shooting were included in the models as covariates. Sexual orientation, ethnicity, mental health, and demographic covariates were collected upon entry into the GfK KnowledgePanel prior to the Pulse nightclub shooting.

## Results

**Figure 1:**
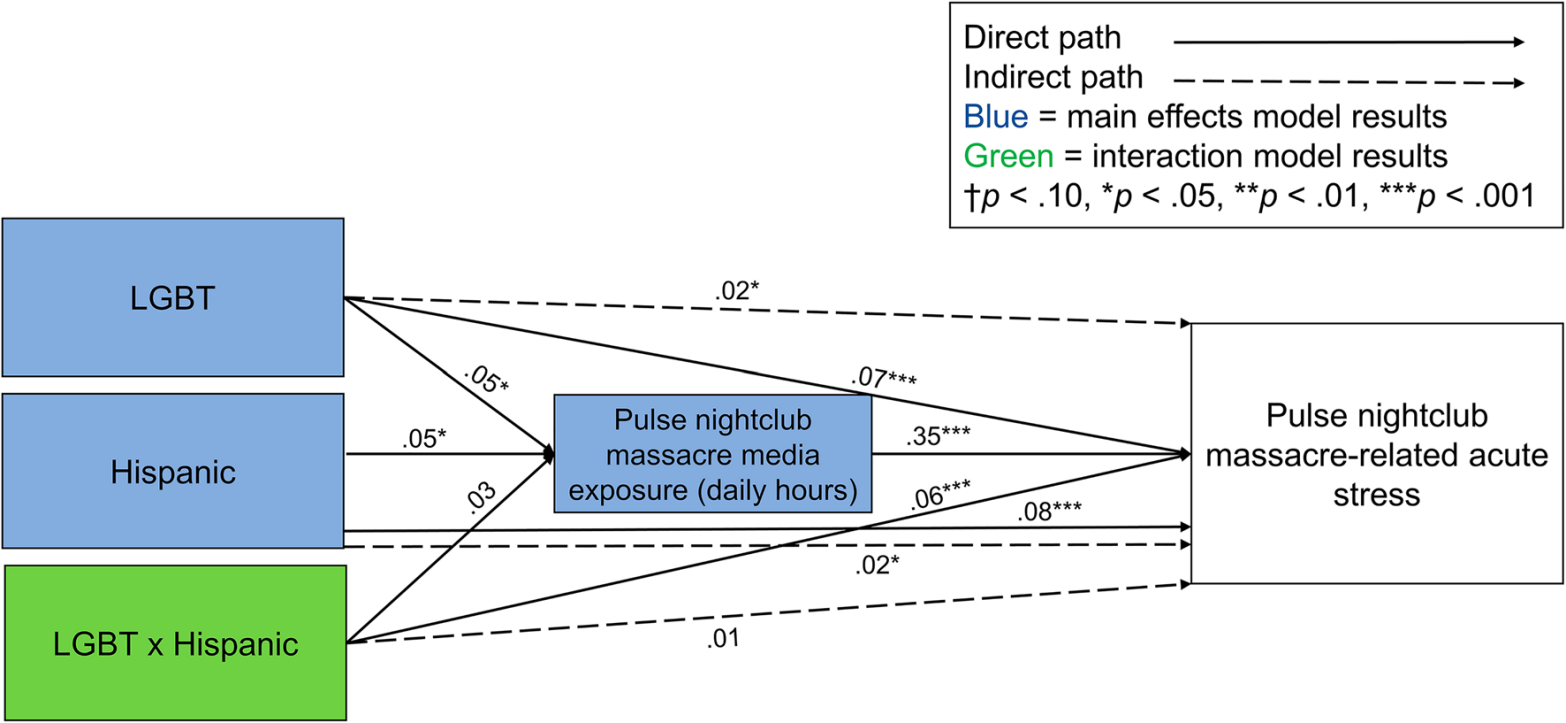
Path models predicting Pulse nightclub massacre-related acute stress from prior self-reported LGBT identity and self-reported Hispanic identity (blue = main effects model), and LGBT × Hispanic identity interaction (green = interaction model). Both models included self-reported average daily hours of Pulse massacre-related media exposure as a mediator and controlled for covariates. Exogenous variables were allowed to correlate, and the models were fit with a full-information maximum likelihood approach.

Sample descriptive statistics and full results are presented in Supplementary Tables 1–5. A path model was constructed predicting acute stress from LGBT and Hispanic identities with indirect paths through self-reported media exposure, controlling for covariates (Fig. 1). The model was fit with a full-information maximum likelihood approach and demonstrated good fit, *χ2* (3) = 5.01, *p* = 0.171, root mean square error of approximation (RMSEA) = 0.012, 95% confidence intervals (CI) [< 0.001, 0.030], *p* = 1.00, comparative fit index (CFI) = 0.998, Tucker-Lewis index (TLI) = 0.976, coefficient of determination (CD) = 0.159 (Supplementary Table 2). Supporting our first hypothesis, participants who identified as LGBT (β = 0.05, standard error (*SE*) = 0.02, 95% CI [0.01, 0.08], *p* = 0.012) or Hispanic (β = 0.05, *SE* = 0.02, 95% CI [0.01, 0.08], *p* = 0.012) reported significantly greater massacre-related media exposure than non-LGBT and non-Hispanics, respectively. This suggests that sharing a social identity with victims of trauma is associated with increased self-reported event-related media exposure. Also as hypothesized, participants who identified as either LGBT (β = 0.07, *SE* = 0.02, 95% CI [0.04, 0.11], *p* < 0.001) or Hispanic (β = 0.08, *SE* = 0.02, 95% CI [0.05, 0.12], *p* < 0.001) reported significantly greater massacre-related acute stress than non-LGBT and non-Hispanics, respectively. This suggests that sharing a social identity with victims of trauma is associated with increased risk of distress.

**Figure 2:**
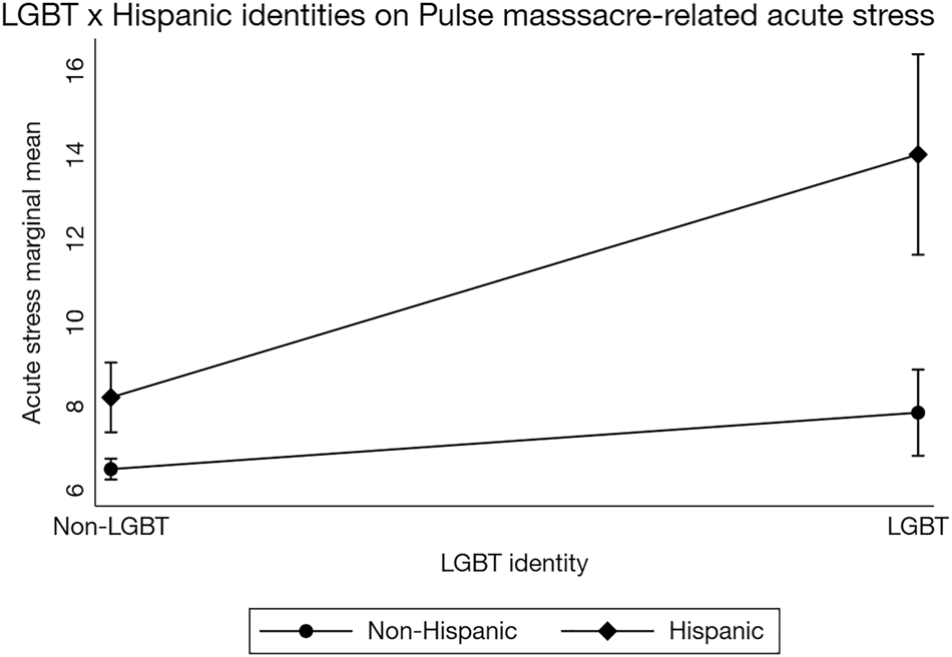
Interaction between LGBT and Hispanic identities on Pulse nightclub massacre-related acute stress, controlling for covariates. Stata 16 does not allow for factor notation using the *sem* command. Thus, this interaction plot was generated using the *regress* command with factor notation among a subset of respondents with complete data (*n* = 3001). The regression equation used to generate this plot was identical to the regression equation used in the full structural equation model. Although the error estimates differ slightly, this simpler model replicates the interaction in the structural equation model. Bars represent 95% confidence intervals. Acute stress ranged from 0 to 56.

The same path model was repeated with the addition of a LGBT × Hispanic identity interaction term (Fig. 1) to capture the potential for additive effects of identifying with both groups. This model demonstrated good fit, *χ2* (3) = 4.94, *p* = 0.176, RMSEA = 0.012, 95% CI [< 0.001, 0.030], *p* = 1.00, CFI = 0.998, TLI = 0.975, CD = 0.165 (Supplementary Table 4). Our second hypothesis received mixed support. The interaction between LGBT and Hispanic identities was not significantly associated with reported Pulse massacre-related media exposure (β = 0.03, *SE* = 0.02, 95% CI [.− 0.01, 0.08], *p* = 0.123). It was, however, associated with acute stress (β = 0.06, *SE* = 0.02, 95% CI [0.02, 0.10], *p* = 0.001; Fig. 2), such that participants who identified as both LGBT and Hispanic reported greater acute stress than those who identified as only one or neither of these identities.

Consistent with prior research^1–11^, there was a significant association between self-reported Pulse massacre-related media exposure and acute stress (β = 0.35, *SE* = 0.02, 95% CI [0.32, 0.38], *p* < 0.001). There were also small, significant indirect effects of LGBT (β = 0.02, *SE* = 0.01, 95% CI [0.003, 0.03], *p* = 0.013) and Hispanic (β = 0.02, *SE* = 0.01, 95% CI [0.004, 0.03], *p* = 0.012; Supplementary Table 3) identities on acute stress through self-reported massacre-related media exposure, supporting our third hypothesis. However, there was no significant indirect effect of LGBT × Hispanic identities on acute stress (β = 0.01, *SE* = 0.01, 95% CI [− 0.003, 0.03], *p* = 0.124; Fig. 2; Supplementary Table 5).

Results demonstrate that sharing social identities with trauma victims is associated with increased risk of event-related media exposure and distress. To conceptually replicate and extend these findings, we conducted a second study investigating the roles of shared social identity and self-reported media exposure in acute stress following a politically-charged event where a personal trauma was publicly shared: the 2018 Dr. Christine Blasey Ford and Judge Brett Kavanaugh Senate hearings prior to confirmation of Judge Kavanaugh to the U.S. Supreme Court.

## 2018 Dr. Blasey Ford and Judge Kavanaugh U.S. Senate hearings

On July 9, 2018, Judge Brett Kavanaugh was nominated to the U.S. Supreme Court to replace retiring Justice Anthony Kennedy. Three weeks later, Dr. Christine Blasey Ford, a psychology professor in California, sent a letter to Senator Dianne Feinstein, a Democrat on the U.S. Senate Judiciary Committee, asserting that Judge Kavanaugh sexually assaulted her in high school. On September 27, 2018, millions of Americans watched and listened as Dr. Blasey Ford and Judge Kavanaugh testified before the U.S. Senate Judiciary Committee. While the hearings were not traumatic in the same way as the Pulse nightclub shooting, where a group was intentionally targeted and the carnage was spread across news media in the immediate aftermath, the hearings involved an individual recounting a past personal traumatic experience in great detail. Moreover, the hearings were widely disseminated live across traditional and social news media platforms, potentially retraumatizing survivors of interpersonal assault. Thus, we consider the hearings to be a publicly shared trauma experienced by a large group of people that was broadcast globally via news media.

This event may have been particularly distressing for people who shared social identities with Dr. Blasey Ford as a woman and/or as a survivor of interpersonal violence. Social identities are more than shared biological characteristics; they also emerge from shared experiences, particularly traumatic ones (e.g., refugees, orphans, veterans). The social identity model of traumatic identity change (SIMTIC) posits that sharing the experiences of trauma can engender a new social identity (e.g., as a victim) in a way that gives meaning to the trauma and an emotional connection to other victims^35,36^. This new social identity, such as a survivor of interpersonal violence, may provide benefits like a shared sense of belonging and connection to others that aids with trauma recovery and resilience ^35^. However, it also provides a new lens through which to perceive and interpret events and feel identity-congruent emotions, with potentially detrimental effects when the trauma-based identity is either targeted or re-experienced. Thus, the widely broadcast details of Dr. Blasey Ford’s assault could lead people who identify as victims of interpersonal violence to experience intrusive thoughts and rumination, which can have downstream consequences for the severity of their posttraumatic stress^48,49^.

More broadly, women may have been at higher risk for distress given that they are more likely to experience sexual assault^50,51^ and the testimonies made such attacks a salient issue^52^. A third salient identity during the hearings was political identity. The hearings occurred during a highly charged standoff between Democrats and Republicans where the successful appointment of Judge Kavanaugh would tip the balance of power in the Supreme Court to favor Republicans, who already controlled the executive and half of the legislative branches of government. Since Dr. Blasey Ford initially came forward with her story to a Democratic Senator, she was portrayed by some Republicans as a political tool used to block their Supreme Court advantage. Hence, Democrats may have been more distressed, not only from identifying with Dr. Blasey Ford as a fellow Democrat re-experiencing a traumatic memory but also because the outcome of the hearings could tip the balance of power by giving the opposing political party a coveted seat on the Supreme Court. We hypothesized that:

1. Participants who share the salient identities of Dr. Blasey Ford (i.e., interpersonal violence victim [weighted *n* = 2298], woman [weighted *n* = 2543], Democrat [weighted* n* = 2248]), would report greater exposure to media coverage about the Senate hearings and greater acute stress than non-victims of interpersonal violence, men, and Republicans, respectively,
2. Participants who identify as both interpersonal violence victim and woman (weighted *n* = 1525), interpersonal violence victim and Democrat (weighted *n* = 1082), or woman and Democrat (weighted *n* = 1175) would report significantly greater Ford and Kavanaugh hearings-related media exposure and greater acute stress than those who identify as one or neither of those identities, and
3. There would be indirect effects of interpersonal violence victim, female, and Democrat identities, and their two-way interactions, on acute stress through self-reported Dr. Blasey Ford and Judge Kavanaugh Senate hearings-related media exposure.

We surveyed a probability-based nationally representative sample of *N* = 4894 from the NORC AmeriSpeak panel, a private survey panel that uses address-based sampling methods to randomly sample and recruit from U.S. households in exchange for compensation. Data collection began the morning of Tuesday October 2, 2018—5 days after Dr. Blasey Ford and Judge Kavanaugh’s testimonies to the U.S. Senate Judiciary Committee and four days before the final confirmation vote—and continued until mid-day Friday October 12, 2018. Participants reported their average daily hours of exposure to media coverage of the hearings using a similar measure as Study 1 and reported their acute stress to the hearings using the five-item Primary Care PTSD screener^53^ for the Diagnostic and Statistical Manual of Mental Disorders-5 (DSM-5)^54^. Participants also reported whether they were a victim of sexual assault, rape, and/or intimate partner violence; upon entry into the AmeriSpeak panel before the Senate hearings respondents had reported their gender, political identity, demographics, and mental health status. A binary victim of interpersonal violence identity variable was created with participants who answered yes to being a victim of sexual assault, rape, and/or intimate partner violence compared to participants who responded no to all three of these. Data were weighted to account for the probability of selection into the sample and differences between our sample and U.S. Census benchmarks.

## Results

**Figure 3:**
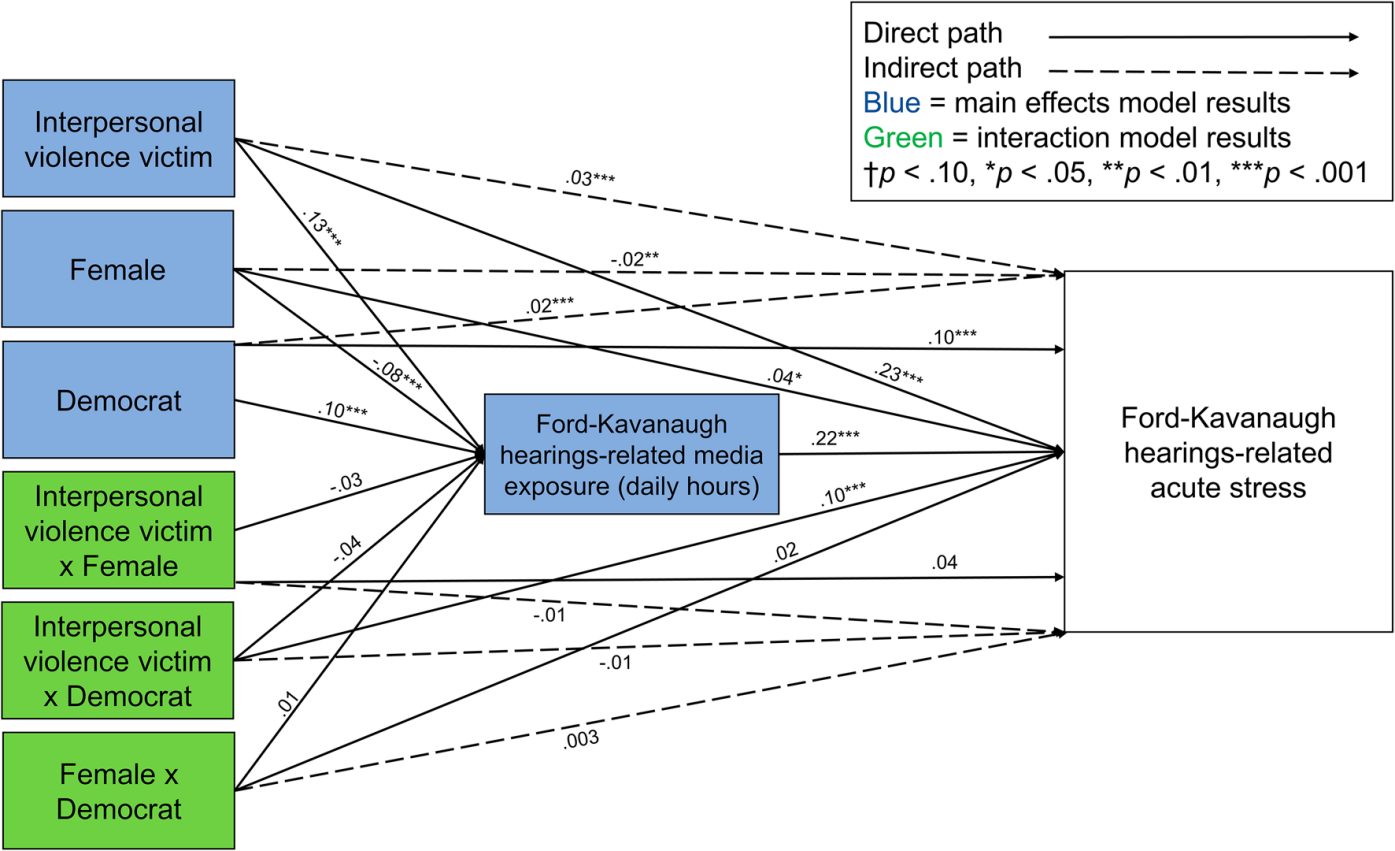
Path models predicting Ford and Kavanaugh hearings-related acute stress from prior self-reported interpersonal violence victim, gender, and political identities (blue = main effects model), and interpersonal violence victim × gender, interpersonal violence victim × political, and  gender × political identity interactions (green = interaction model). Both models included self-reported average daily hours of Ford and Kavanaugh hearings-related media exposure as a mediator and controlled for covariates. Exogenous variables were allowed to correlate, and the models were fit with a full-information maximum likelihood approach.

Sample descriptive statistics and full results are presented in the Supplementary Tables 6–10. A path model was constructed predicting acute stress from victim of interpersonal violence, gender, and political party identities with indirect paths through self-reported media exposure, controlling for covariates (Fig. 3). The model was fit with a full-information maximum likelihood approach, CD = 0.191 (Supplementary Table 7). Supporting our first hypothesis, participants who shared Dr. Blasey Ford’s identity as a victim of interpersonal violence (β = 0.13, *SE* = 0.02, 95% CI [0.08, 0.18], *p* < 0.001) or as a Democrat (β = 0.10, *SE* = 0.02, 95% CI [0.05, 0.14], *p* < 0.001) reported significantly greater exposure to media coverage of the hearings than non-interpersonal violence victims and Republicans, respectively. Counter to our hypothesis, women reported significantly less Ford and Kavanaugh hearings media exposure than men (β = − 0.08, *SE* = 0.02, 95% CI [− 0.13, − 0.04], *p* < 0.001). However, supporting the first hypothesis, participants who identified as a victim of interpersonal violence (β = 0.23, *SE* = 0.02, 95% CI [0.19, 0.27], *p* < 0.001), woman (β = 0.04, *SE* = 0.02, 95% CI [0.01, 0.08], *p* = 0.024), or Democrat (β = 0.10, *SE* = 0.02, 95% CI [0.06, 0.14], *p* < 0.001) reported significantly greater Ford and Kavanaugh hearings-related acute stress than non-interpersonal violence victims, men, and Republicans, respectively.

**Figure 4:**
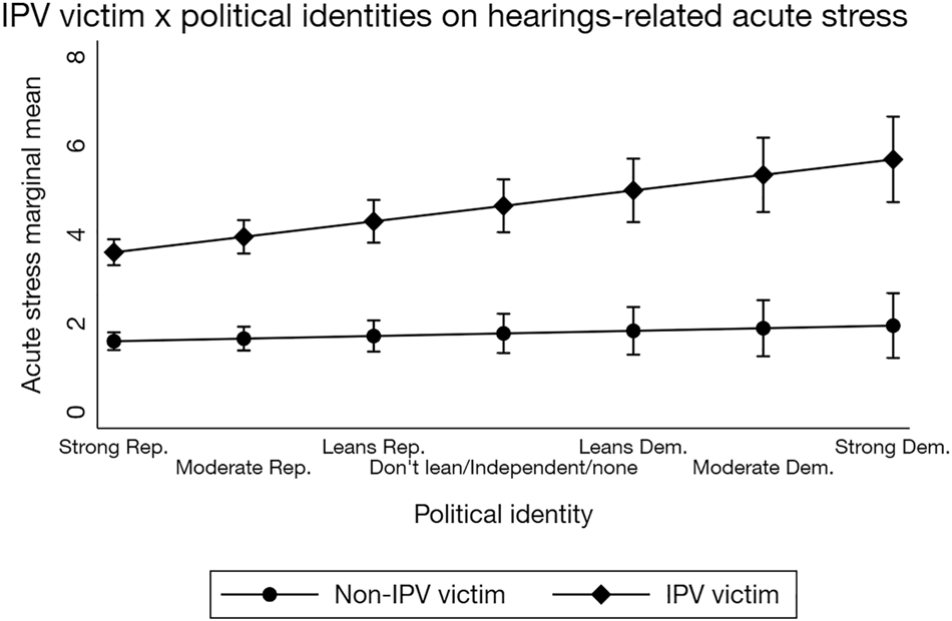
Interaction between interpersonal violence victim and political identities on Ford and Kavanaugh hearings-related acute stress, controlling for covariates. Exogenous variables were allowed to correlate, and the model was fit with a full-information maximum likelihood approach. Stata 16 does not allow for factor notation using the *sem* command. Thus, the interaction plot was generated using the regress command with factor notation and weighting among a subset of respondents with complete data (*n* = 4828). The regression equation used to generate this plot was identical to the regression equation used in the full structural equation model. Although the error estimates differ slightly, this simpler model replicates the interaction in the structural equation model. Rep. = Republican, Dem. = Democrat, IPV = interpersonal violence. Bars represent 95% confidence intervals. Acute stress ranged from 0 to 20.

The same path model was repeated with the addition of interpersonal violence victim × gender, interpersonal violence victim × political party identity, and gender × political party identity interaction terms, using a full-information maximum likelihood approach, CD = 0.199. (Fig. 3; Supplementary Table 9). Our second hypothesis received mixed support. None of the dual identity interactions were significantly associated with self-reported Ford and Kavanaugh hearings-related media exposure. Of the three dual identity interactions, interpersonal violence victim × Democrat was significantly associated with acute stress (β = 0.10, *SE* = 0.03, 95% CI [0.05, 0.16], *p* < 0.001; Fig. 4) such that sharing these two identities with Dr. Blasey Ford was associated with greater distress than sharing a single or neither identity.

Consistent with prior research^1–11,43^ and Study 1, self-reported exposure to media coverage of the Ford and Kavanaugh hearings was significantly associated with acute stress (β = 0.22, *SE* = 0.02, 95% CI [0.17, 0.27], *p* < 0.001), and there were small, significant indirect effects of interpersonal violence victim (β = 0.03, *SE* = 0.01, 95% CI [0.02, 0.04], *p* < 0.001), male gender (ꞵ = − 0.02, *SE* = 0.01, 95% CI [− 0.03, − 0.01], *p* = 0.001), and Democrat (β = 0.02, *SE* = 0.01, 95% CI [0.01, 0.03], *p* < 0.001; Supplementary Table 8) identities on acute stress through self-reported media exposure. Counter to the third hypothesis, there were no significant indirect effects of the three dual identity interaction terms (Supplementary Table 10).

Results from Study 2 partially replicate Study 1 in that sharing a social identity with victims of trauma was associated with increased self-reported event-related media exposure and distress. However, results about sharing Dr. Blasey Ford’s gender identity were more nuanced. Men reported greater hearings-related media exposure than women, and there was a significant indirect effect of male identity on acute stress through reported media exposure. Nonetheless, results support the proposition that sharing a social identity with trauma victims is associated with both increased self-reported exposure to trauma-related media coverage and acute stress, including in contexts where personally experienced traumas are publicly shared via the media.

## Discussion

In two different contexts where traumatic experiences were broadcast widely by news media, people who shared social identities with victims reported more event-related media exposure and distress. Results from both studies reported here are consistent with prior findings that self-reported exposure to media coverage of trauma^1–11,43^, and sharing social identities with trauma victims^22–28,31–36^, are associated with increased distress. Specifically, participants in our national sample in Study 1 who shared either LGBT or Hispanic identities with the Pulse nightclub shooting victims reported greater shooting-related media exposure and greater acute stress than people who shared neither identity. There was also an indirect effect of shared social identity on acute stress through self-reported media exposure, suggesting that media exposure explained, in part, the association between shared social identity with the victims and distress. These findings were replicated in a different context that similarly involved widespread media dissemination of a traumatic experience. During the Dr. Blasey Ford and Judge Kavanaugh U.S. Senate hearings, participants who shared any one of three social identities (as a woman, survivor of interpersonal violence, and Democrat) with a woman who recounted her traumatic experience live in great detail also reported greater acute stress. Results for self-reported media exposure were largely supported, where sharing four out of the five social identities examined across the two studies significantly predicted greater self-reported event-related media exposure.

Although we found consistent evidence for the hypothesized association between sharing a single social identity with trauma victims and acute stress, results were less clear for sharing dual social identities. In the aftermath of the Pulse nightclub shooting, sharing both LGBT and Hispanic identities significantly predicted acute stress. However, of the three social identity interactions tested in the Ford and Kavanaugh hearings study, only the interaction between interpersonal violence victim and Democrat significantly predicted acute stress. In both studies, there was no association between sharing dual social identities and self-reported media exposure.

One potential reason for these mixed dual identity results is because these were qualitatively different events. The Pulse nightclub shooting was a collective trauma where an individual targeted an underrepresented group to instill terror. The most salient identities of the attack victims were clear because they were related to the motive behind the attack. The intentionality behind the attack is important because it erodes trust in others among social ingroup members of the victims, leaving them feeling disconnected and betrayed^27,55^.

On the other hand, the Dr. Blasey Ford and Judge Kavanaugh hearings involved widespread media coverage of a personal traumatic event where a survivor of interpersonal violence recounted her traumatic experience amidst a highly polarized political environment. Indeed, although this event consisted of an individual recounting her traumatic experience in great detail, it is a trauma that is commonly experienced across the world, particularly by women. According to the World Health Organization (WHO), approximately 1 in 3 women worldwide have been subjected to either physical and/or sexual intimate partner or non-partner violence in their lifetime^56^. In the U.S., data from the Centers for Disease and Control and Prevention (CDC) National Intimate Partner and Sexual Violence Survey (NISVS) show that about 1 in 3 women, and 1 in 4 men, reported having experienced severe physical violence from an intimate partner in their lifetimes^56^. Moreover, about 1 in 5 women and 1 in 13 men reported having experienced sexual violence by an intimate partner^57^. Thus, publicly and widely sharing the details of Dr. Blasey Ford’s assault live on global news created a moment of shared trauma-related experience, especially for those who shared an identity as a survivor of interpersonal violence.

Moreover, with major political implications at stake, the narrative surrounding the facts of Dr. Blasey Ford’s assault became polarized, leading to politicized perceptions of victimhood. For instance, as Judge Kavanaugh expressed outrage during his testimony, he claimed to be the victim of a false sexual assault allegation—a political weapon to block his appointment to the Supreme Court^58^. By blurring the lines of gender victimhood with an event where the trauma is recounted and the facts more easily debated (as opposed to seeing news media scenes of ongoing death and destruction), the cues for which gender should feel most threatened were less clear (e.g.,^52^). What was clear, however, was that the motivation behind the hearings was directly tied to interpersonal violence, making interpersonal violence survivorship a salient identity. It was also clear that if Judge Kavanaugh was successfully appointed to the Supreme Court, the balance of power would shift in favor of Republicans, making the threat that the outcome of the hearings poses to Democrats salient. Greater attention to psychological experiences engendered by multiple impacted social identities can inform public health research on vulnerable subpopulations^58^.

More broadly, our findings suggest that people use media in line with their social identities. There is scant research on how social identities drive media engagement, and how such engagement might influence identification with social groups. Some have theorized that people use media that portray their social identities in a positive light^60,61^. However, our findings suggest engaging with identity-related media may be detrimental in the context of trauma. This is especially true considering that exposure to trauma-related media can further amplify distress^1–11^, a finding we replicated in both studies.

Our results have implications for media engagement and public health. Media organizations should consider limiting sensational coverage of traumatic events, as it may promote distress among people who identify with victims. Likewise, the general public should be made aware of the negative repercussions of trauma-related media exposure and the increased likelihood of engaging with it when it is self-relevant. Moreover, to protect vulnerable populations following trauma, future research should test the efficacy of targeting recovery messages and treatments like group-based emotion regulation^62^ toward members of victims’ ingroups.

## Limitations

Although identities in both studies were measured before each event, the correlational nature of our results restricts our ability to claim causality. Thus, we are unable to discern whether identification with trauma victims motivates exposure to event-related media coverage or exposure to event-related media coverage motivates identification with victims (e.g.,^63^). The relation may also be bidirectional, whereby those who identify with trauma victims experience distress, which in turn motivates attention to media, which further increases identification and distress^3^. Additional research is needed to better understand these links and potential interventions that can ameliorate negative consequences for public health.

Our studies were also limited in not *directly* measuring the degree to which participants identify with the victims. Rather, we inferred identification from demographic reporting before both events, which may have led to the small effects we observed. Small effects, however, can be impactful when repeated over time^64^. For instance, results from the present studies suggest that exposure to media coverage of a single instance of police brutality involving a Black American victim may have a small, yet significant, identification effect on Black Americans’ mental health around the country^65,66^, but the repetition of such events may have an even larger and longer-lasting negative psychological impact. This contradicts research suggesting that minority identification can buffer the negative effects of prejudice^30^. Future research should explore the longitudinal effects of shared social identities on media exposure and distress in the context of publicly shared traumas.

Additionally, we did not explore how some shared identities are more easily hidden (e.g., LGBT) than others (e.g., Hispanic), which may impact quality of life^67^. Our results showed nearly equal acute stress effect sizes between concealable (LGBT, interpersonal violence victim, political affiliation) and non-concealable (race/ethnicity, gender) identities. However, the concealability of shared social identities might matter for how quickly people respond to traumas spread publicly via news media (e.g., victims’ identities may be more immediately obvious as an event occurs when the identities are non-concealable). Future research should investigate whether this difference affects psychological outcomes following traumatic events.

We also note that our measure of event-related media exposure was self-report, which may be limited by biased time estimation. It is possible that participants either misremembered how much time they spent exposed to event-related media, or that their personal motivations to either engage or not with news coverage of the event biased how much time they thought they spent exposed to such media coverage. For instance, someone morally outraged by aspects of the Dr. Blasey Ford and Judge Kavanaugh hearings might overestimate how much time they spent exposed to hearings-related media coverage, or someone apathetic to the event might underestimate such exposure. Though this remains a possibility, our analyses controlled for several variables that may lead to spurious correlations between media exposure and acute stress, such as prior mental health diagnosis, employment status, education, and income.

Another limitation of our findings is that we may have been underpowered to detect statistical significance, particularly in Study 1. Though the sample was large with over 4000 participants, there were only *n* = 50 who identified (prior to the study) as both LGBT and Hispanic. Despite the low subsample size, we still observed a significant dual identity interaction on Pulse massacre-related acute stress. Yet, lack of statistical power may explain why there was no significant interaction on self-reported massacre-related media exposure and no significant dual identity indirect effects on acute stress through reported media exposure. That we replicated this pattern of a dual identity interaction on acute stress but not reported media exposure nor with indirect effects in Study 2, where we had much larger dual identity subsamples (*ns* = 1082–1525), suggests that power may not have been an issue in Study 1. Future research should consider using more targeted quota sampling of underrepresented dual identities to improve statistical power in investigating the role of shared social identities in response to publicly shared traumas.

A final limitation of Study 1 is that the sample completed the survey from 5 days to approximately one month after the Pulse massacre. It is possible that, as the massacre got farther away from recent memory, people would report less stress and less massacre-related media exposure. To address this limitation, we re-ran the models including survey completion date as a covariate and found that there was no significant association with either self-reported media exposure or acute stress (*p*s > 0.100). Indeed, media coverage of the attack, which was the deadliest mass shooting in U.S. history at the time, was extensive and ongoing, with major news outlets providing updates and analysis on the investigation, the victims, and the response from the community and political leaders. Additionally, the story generated significant debate and discussion about issues such as gun control, LGBT rights, and terrorism, which further contributed to its continued coverage in the media. While the intensity of the coverage gradually decreased over time, news outlets continued to report on the aftermath of the shooting, including the trial of the shooter's wife, the ongoing investigation into the incident, and the community's efforts to recover and heal from the tragedy. Time since event was less likely an issue for the Dr. Blasey Ford and Judge Kavanaugh hearings since the survey was administered in the days following the hearings and prior to the final Senate confirmation vote.

## Conclusions

Belonging to social groups can be beneficial in providing resources and social support when experiencing stress^30,34,68,69^, and this benefit can be accessed today from geographically remote locations. However, the transmission of ingroup members’ experiences can also be detrimental, particularly when such experiences are traumatic. We demonstrate that sharing social identities with trauma victims is associated with self-reported trauma-related media exposure and distress. Thus, social identities may be a double-edged sword when the trauma is spread widely through the media in that they engender a sense of belonging and meaningful connections with others, but also a shared sense of distress when the identities are targeted and the traumatic experience is broadcast widely.

## Materials and methods

### 2016 Orlando Pulse nightclub massacre

Participants were recruited as the sixth wave of a longitudinal population-based study investigating Americans’ psychological reactions to collective traumas, which began shortly after the Boston Marathon bombings^11^. The initial sample was drawn from the GfK KnowledgePanel (with oversampling in metropolitan Boston and New York City), a nationally representative panel of U.S. residents recruited using address-based sampling methods. The survey was fielded from June 17-July 22, 2016 to 6098 people, of whom 4822 responded during the fielding period and 4675 provided usable data, yielding a 76.66% completion rate. Despite recruiting a national sample, sampling weights were not applied in Study 1 because the U.S. Census does not track sexual orientation nor transgender identity and thus does not have a population benchmark for comparison. Participants received free internet or compensation. All methods were carried out in accordance with relevant guidelines and regulations and all procedures were reviewed and approved by the Institutional Review Board of the University of California, Irvine. All participants provided informed consent.

### Measures

#### Acute stress

Acute stress symptoms related to the Pulse nightclub massacre were assessed using the Acute Stress Disorder Scale 5^47^, which measured the frequency of experiencing 14 symptoms of acute stress from the Diagnostic and Statistical Manual of Mental Disorders, 5th edition (DSM-5)^54^ related to the Pulse nightclub massacre (e.g., “Do you try to avoid thinking about the Orlando mass shooting?”). Participants indicated the extent to which they experienced symptoms using a 5-point Likert-type scale ranging from *not at all* (0) to *a great deal* (4). Responses were summed, ranging from 0 to 56, α = 0.88.

#### Self-reported media exposure

Participants reported the average daily number of hours they watched or listened to media coverage about the Orlando mass shooting in the days after it happened. Using a checkbox grid format, participants indicated the number of hours from *0* to *11* + for five media sources, including television, radio, online news sources (e.g., CNN, Yahoo, NYTimes.com, etc.), pictures and/or videos on social media (e.g., Facebook, Twitter, etc.), and news or text updates on social media (Twitter, Reddit, etc.). This format allowed us to capture simultaneous modes of event-related news media exposure, such as scrolling through social media while watching television. Total average daily hours of media exposure were summed across all five media types to create an index of media exposure. Hours were capped at 18 (3 standard deviations above the mean) to account for outliers.

#### LGBT identity

Upon entry into the GFK KnowledgePanel, participants reported several demographic characteristics, including transgender identity and sexual orientation. Participants indicated whether they are cisgender (*n* = 4388) or transgender (*n* = 41), then identified as straight (i.e., heterosexual; *n* = 4181), gay or lesbian (*n* = 127), bisexual (*n* = 88) or something else (i.e., other; *n* = 39). A dichotomous variable was created comparing cisgender heterosexuals (non-LGBT, *n* = 4125) to those who identified as transgender, gay, lesbian, bisexual, or something else (LGBT, *n* = 287). Subsample sizes differ from the sample total due to missing data. To account for this, our analysis used full-information maximum likelihood estimation.

#### Hispanic identity

Participant ethnicity was also collected upon entry into the GfK KnowledgePanel before the Pulse nightclub attack. Participants reported whether they were Black non-Hispanic (*n* = 387), Hispanic (*n* = 478), other non-Hispanic (*n* = 308), or White non-Hispanic (*n* = 3502). Ethnicity was recoded into two categories: non-Hispanic (*n* = 4197) and Hispanic (*n* = 478).

#### Other demographics

Age, gender, education, household income, employment status, marital status, strength of political party identity and U.S. Census Bureau designated geographic region were collected upon entry to the KnowledgePanel prior to the Pulse nightclub massacre.

#### Mental health diagnoses

Mental health status was assessed as part of a comprehensive health assessment upon entry into the GfK KnowledgePanel. Participants were given a list of physical and mental health ailments and indicated whether they had ever been diagnosed by a physician for each. Responses were recoded to three levels: no diagnosis of anxiety or depression, diagnosis of anxiety *or* depression, and diagnoses of both anxiety *and* depression.

#### Direct Pulse massacre exposure

Participants reported whether they or someone they knew was at or near the site of the shooting as either *no* or *yes*.

#### Covariates

Covariates included age, gender, education, household income, employment status, marital status, strength of political party identity, U.S. Census Bureau designated geographic region, prior self-reported mental health diagnoses, and direct exposure to the Pulse nightclub massacre.

### Analytic strategy

Statistical analyses were conducted using Stata version 16^70^. Summary scores were created for self-reported Pulse massacre-related media exposure and acute stress to account for variability^71^, and categorical variables were dummy coded. Two path models were constructed, one with LGBT identity and Hispanic identity main effects and another adding an LGBT × Hispanic identity interaction term, predicting acute stress, controlling for covariates. Self-reported media exposure was specified as a mediator between predictor variables and acute stress. Exogenous variables were free to correlate, and the models were fit with a full-information maximum likelihood approach to account for missing data.

## 2018 Dr. Blasey Ford and Judge Kavanaugh U.S. Senate hearings

Participants in Study 2 were recruited from the NORC AmeriSpeak panel as part of a study investigating the psychological impact of the Dr. Blasey Ford and Judge Kavanaugh hearings on Americans. Data collection began the morning of October 2, 2018—5 days after Dr. Blasey Ford and Judge Kavanaugh’s testimonies to the U.S. Senate Judiciary Committee and four days before the final confirmation vote—and continued until mid-day October 12, 2018. Among the 8237 panelists invited to participate, 4894 completed the survey during the fielding period, yielding a 59.41% completion rate. Data were weighted to account for probability of selection into the AmeriSpeak panel and demographic differences in our sample compared to U.S. Census benchmarks. All participants were compensated for their time. All methods were carried out in accordance with relevant guidelines and regulations and all procedures were reviewed and approved by the Institutional Review Board of the University of California, Irvine. All participants provided informed consent.

### Measures

#### Acute stress

Acute stress symptoms related to the Dr. Blasey Ford and Judge Kavanaugh hearings were assessed using the Primary Care PTSD screener for the DSM-5^51^ with a modified scale format. Participants indicated the extent to which they experienced five symptoms of acute stress as a result of the news story about the Senate Hearings (e.g., “Had nightmares about sexual or interpersonal violence or thought about it when you did not want to?”) using a 5-point Likert-type scale from *never* (0) to *all the time* (4). Responses were summed, ranging from 0 to 20, α = 0.87.

#### Self-reported media exposure

Daily hours of exposure to hearings-related media coverage were measured using similar items and format as Study 1. Participants indicated the number of hours, on average, in the past week they spent reading, watching, and/or listening to media coverage of the Judge Kavanaugh and Dr. Ford news story from *0* to *11*+ for television, radio or podcasts, online news sources (CNN, Yahoo, NYTimes.com, etc.), news or text updates on social media (Twitter, Facebook, Reddit, etc.), and print news sources (newspaper, magazines). Hours were summed across the five media types and capped at 25 (3 standard deviations above the mean) to account for outliers.

#### Interpersonal violence victim identity

Participants indicated whether they were ever a victim of sexual assault, rape, or intimate partner violence. Participants who answered yes to any of the following three items: “Has anyone ever touched or felt private areas of your body under force of threat or forced you to touch or feel someone else’s private areas?” (weighted *n* = 1491), “Have you ever had sexual relations under force or threat?” (weighted *n* = 832), and “Have you ever been hit or pushed by a partner or spouse?” (weighted *n* = 1553), were coded as a victim of interpersonal violence (weighted *n* = 2298), and participants who answered no to all three of these were coded as a non-victim (weighted *n* = 2529).

#### Gender identity

Participants indicated their gender as either *male* (0) or *female* (1) upon entry to the AmeriSpeak panel.

#### Strength of political identity

Participants indicated the strength of their political party identity from *strong Democrat* (1) to *don’t lean/Independent/none* (4) to *strong Republican* (7). The item was reverse scored for easier interpretation of results so that higher scores indicate stronger identification as Democrat.

#### Other demographics

Ethnicity, age, household income, education, employment status, marital status, U.S. Census Bureau designated geographic region were collected upon entry to the AmeriSpeak panel, prior to the Ford and Kavanaugh hearings. These variables were included as covariates in analyses.

### Analytic strategy

Statistical analyses were conducted using Stata version 16^70^. Summary scores were created for self-reported hearings-related media exposure and acute stress to account for variability^71^, and categorical variables were dummy coded. Two path models were constructed, one with interpersonal violence victim, gender, and political identity main effects and another adding two-way interactions among these three variables, predicting acute stress, controlling for covariates (the significance of the results does not change when the model is repeated with each interaction term included on its own). Self-reported media exposure was specified as a mediator between predictor variables and acute stress. Exogenous variables were free to correlate, and the models were fit with a full-information maximum likelihood approach. Statistical weights were applied to account for selection into the AmeriSpeak panel and differences between our sample and U.S. Census benchmarks. Model fit was based on the CD, which is analogous to *R*^*2*^ in linear regression and is the percentage of variance explained by the model. CD was used because estimates of other common fit indices, such as CFI, are often biased in complex weighted survey analysis^72^.

## Supplementary Information

Supplementary Information.

## Data Availability

Data, code and materials generated during and/or analyzed for both studies are available from the corresponding author upon request.
