# Supplementary material for: Shared social identity and media transmission of trauma
Source: Sci Rep. 2023 Jul 18;13:11609. doi: 10.1038/s41598-023-33898-2 (PMC10354080; doi:10.1038/s41598-023-33898-2)
Supplement: Supplementary file 1 — Supplementary Information. [file 41598_2023_33898_MOESM1_ESM.pdf]

Supplementary Information

for

Shared social identity and media transmission of trauma

Daniel P. Relihan

Nickolas M. Jones

E. Alison Holman

Roxane Cohen Silver

Corresponding author: Roxane Cohen Silver, Ph.D., Department of Psychological Science, 4201 Social & Behavioral Sciences Gateway, University of California, Irvine, CA 92697-7085. Email: rsilver@uci.edu.

Supplementary Table 1. Study 1 Pulse nightclub massacre sample descriptive statistics. $N = 4,675$

Variable	*n* (%)	Mean (*SD*)	95% CI	Range
Age		52.72 (16.92)	52.23, 53.20	20, 96
Gender				
Male	2,149 (45.97)			
Female	2,526 (54.03)			
Education				
Less than high school	238 (5.09)			
High school	1,143 (24.45)			
Some college	1,356 (29.01)			
Bachelor's degree or higher	1,938 (41.45)			
Household income		3.55 (2.02)	3.49, 3.61	1, 8
Less than $24,999	743 (15.89)			
$25,000 to $49,999	1,000 (21.39)			
$50,000 to $74,999	854 (18.27)			
$75,000 to $99,999	721 (15.42)			
$100,000 to $124,999	551 (11.79)			
$125,000 to $149,999	301 (6.44)			
$150,000 to $174,999	191 (4.09)			
$175,000 or more	314 (6.72)			
Employment status				
Not working (temp layoff, retired, disabled, other)	2,004 (42.87)			
Working (self-employed, as a paid employee)	2,671 (57.13)			
Marital status				
Married / Cohabitating	2,931 (62.70)			
Widowed / Divorced / Separated	862 (18.44)			
Never married / Single	882 (18.87)			
Strength of political party affiliation		4.31 (2.03)	4.25, 4.37	1, 7
Strong Republican	540 (11.92)			
Not strong Republican	490 (10.82)			
Leans Republican	845 (18.66)			
Undecided / Independent / other	201 (4.44)			
Leans Democrat	926 (20.45)			
Not strong Democrat	641 (14.15)			

Strong Democrat	886 (19.56)			
U.S. Census Bureau geographic region				
Northeast	2,076 (44.41)			
Midwest	725 (15.51)			
South	1,172 (25.07)			
West	702 (15.02)			
Prior self-reported mental health diagnoses				
Neither anxiety nor depression	3,784 (80.94)			
Anxiety or depression	563 (12.04)			
Anxiety and depression	328 (7.02)			
Direct exposure to Pulse nightclub massacre				
No	2,896 (90.53)			
Yes	303 (9.47)			
LGBT identity				
Non-LGBT	4,125 (93.50)			
LGBT	287 (6.50)			
Hispanic identity				
Non-Hispanic	4,197 (89.78)			
Black, non-Hispanic	387 (8.28)			
Other, non-Hispanic	308 (6.59)			
White, non-Hispanic	3,502 (74.91)			
Hispanic	478 (10.22)			
LGBT x Hispanic identities				
Non-LGBT, non-Hispanic	3,724 (84.41)			
LGBT, non-Hispanic	237 (5.37)			
Non-LGBT, Hispanic	401 (9.09)			
LGBT, Hispanic	50 (1.13)			
Self-reported daily hours of Pulse nightclub massacre media exposure		3.21 (3.60)	3.08, 3.34	0, 18+
Self-reported Pulse nightclub massacre-related acute stress		6.82 (7.38)	6.57, 7.08	0, 56

Note: *SD* = standard deviation; CI = confidence intervals (lower limit, upper limit); Range = minimum, maximum; LGBT = lesbian, gay, bisexual, transgender; Numbers may vary due to missing data.

Supplementary Table 2. Study 1 Pulse nightclub massacre structural equation model standardized direct main effects. $N = 4{,}675$

	Path to media exposure		Path to acute stress	
Variable	β (95% CI)	*p*	β (95% CI)	*p*
Age	-.03 (-.07, .02)	.206	.04 (.01, .08)	.024
Gender (comparison = male)				
Female	.002 (-.03, .04)	.891	.06 (.03, .10)	< .001
Education (comparison = less than high school)				
High school	-.09 (-.18, -.004)	.041	-.04 (-.12, .04)	.334
Some college	-.15 (-.24, -.05)	.002	-.07 (-.15, .02)	.111
Bachelor's degree or higher	-.18 (-.28, -.07)	.001	-.06 (-.16, .03)	.173
Household income	-.04 (-.08, .002)	.062	-.08 (-.12, -.05)	< .001
Employment status (comparison = not working)				
Working	-.01 (-.05, .03)	.476	-.01 (-.04, .03)	.755
Marital status (comparison = married / cohabitating)				
Widowed / Divorced / Separated	-.01 (-.05, .03)	.709	.02 (-.02, .05)	.331
Never married / Single	.07 (.03, .11)	< .001	-.01 (-.05, .02)	.454
Strength of political party affiliation (higher = stronger Democrat)	.06 (.02, .09)	.001	.03 (-.003, .06)	.074
U.S. Census-designated geographic region (comparison = Northeast)				
Midwest	--	--	-.05 (-.08, -.01)	.007
South	--	--	-.02 (-.05, .02)	.364
West	--	--	-.01 (-.04, .03)	.646
Prior self-reported mental health diagnoses (comparison = neither anxiety nor depression)				
Anxiety or depression	.03 (-.001, .07)	.059	.08 (.04, .11)	< .001
Anxiety and depression	.01 (-.02, .05)	.543	.17 (.14, .20)	< .001
Direct exposure to Pulse nightclub massacre (comparison = no)				
Yes	.16 (.12, .19)	< .001	.09 (.06, .12)	< .001
LGBT identity (comparison = non-LGBT)				
LGBT	.05 (.01, .08)	.012	.07 (.04, .11)	< .001
Hispanic identity (comparison = non-Hispanic)				
Hispanic	.05 (.01, .08)	.012	.08 (.05, .12)	< .001
Self-reported daily hours of Pulse nightclub massacre media exposure	--	--	.35 (.32, .38)	< .001
Constant	1.13 (.85, 1.40)	< .001	.50 (.24, .76)	< .001

Model statistics	$\chi^2(3) = 5.01$, $p = .171$<br>CD = .159<br>RMSEA = .012, 95% CI[< .001, .030], $p = 1.00$<br>CFI = .998<br>TLI = .976<br>AIC = 161,884.52<br>BIC = 163,348.67

Note: CI = confidence intervals (lower limit, upper limit); LGBT = lesbian, gay, bisexual, transgender; -- = no path; CD = coefficient of determination; RMSEA = root mean square error of approximation; CFI = comparative fit index; TLI = Tucker-Lewis index; AIC = Akaike information criterion; BIC = Bayesian information criterion.

Supplementary Table 3. Study 1 Pulse nightclub massacre structural equation model standardized indirect effects on acute stress through media exposure. $N = 4{,}675$

Variable	β (95% CI)	*p*
Age	-.01 (-.02, .01)	.207
Gender (comparison = male)		
Female	.001 (-.01, .01)	.891
Education (comparison = less than high school)		
High school	-.03 (-.06, -.001)	.043
Some college	-.05 (-.08, -.02)	.002
Bachelor's degree or higher	-.06 (-.10, -.03)	.001
Household income	-.01 (-.03, .001)	.062
Employment status (comparison = not working)		
Working	-.01 (-.02, .01)	.477
Marital status (comparison = married / cohabitating)		
Widowed / Divorced / Separated	-.003 (-.02, .01)	.709
Never married / Single	.02 (.01, .04)	.001
Strength of political party affiliation (higher = stronger Democrat)	.02 (.01, .03)	.002
Prior self-reported mental health diagnoses (comparison = neither anxiety nor depression)		
Anxiety or depression	.01 (-.001, .02)	.060
Anxiety and depression	.004 (-.01, .02)	.543
Direct exposure to Pulse nightclub massacre (comparison = no)		
Yes	.05 (.04, .07)	< .001
LGBT identity (comparison = non-LGBT)		
LGBT	.02 (.003, .03)	.013
Hispanic identity (comparison = non-Hispanic)		
Hispanic	.02 (.004, .03)	.012

Note: CI = confidence intervals (lower limit, upper limit); LGBT = lesbian, gay, bisexual, transgender.

Supplementary Table 4. Study 1 Pulse nightclub massacre structural equation model standardized LGBT x Hispanic interaction direct main effects. *N* = 4,675

	Path to media exposure		Path to acute stress	
Variable	β (95% CI)	*p*	β (95% CI)	*p*
Age	-.03 (-.07, .02)	.208	.04 (.01, .08)	.024
Gender (comparison = male)				
Female	.003 (-.03, .04)	.866	.07 (.03, .10)	< .001
Education (comparison = less than high school)				
High school	-.09 (-.18, -.004)	.041	-.04 (-.12, .04)	.325
Some college	-.15 (-.24, -.05)	.002	-.07 (-.15, .02)	.111
Bachelor's degree or higher	-.18 (-.28, -.07)	.001	-.06 (-.16, .03)	.166
Household income	-.04 (-.08, .002)	.066	-.08 (-.12, -.04)	< .001
Employment status (comparison = not working)				
Working	-.01 (-.05, .03)	.495	-.004 (-.04, .03)	.795
Marital status (comparison = married / cohabitating)				
Widowed / Divorced / Separated	-.01 (-.05, .03)	.704	.02 (-.02, .05)	.337
Never married / Single	.07 (.03, .11)	< .001	-.01 (-.05, .02)	.517
Strength of political party affiliation (higher = stronger Democrat)	.06 (.02, .09)	.001	.03 (-.001, .06)	.059
U.S. Census-designated geographic region (comparison = Northeast)				
Midwest	--	--	-.05 (-.08, -.01)	.007
South	--	--	-.02 (-.05, .02)	.319
West	--	--	-.01 (-.04, .02)	.618
Prior self-reported mental health diagnoses (comparison = neither anxiety nor depression)				
Anxiety or depression	.03 (-.001, .07)	.060	.08 (.04, .11)	< .001
Anxiety and depression	.01 (-.02, .05)	.508	.17 (.14, .20)	< .001
Direct exposure to Pulse nightclub massacre (comparison = no)				
Yes	.16 (.12, .19)	< .001	.09 (.06, .12)	< .001
LGBT identity (comparison = non-LGBT)				
LGBT	.03 (-.004, .07)	.082	.05 (.02, .09)	.005
Hispanic identity (comparison = non-Hispanic)				
Hispanic	.04 (-.001, .08)	.054	.07 (.03, .10)	< .001
LGBT x Hispanic identities	.03 (-.01, .08)	.123	.06 (.02, .10)	.001

Self-reported daily hours of Pulse nightclub massacre media exposure	--	--	.35 (.32, .38)	< .001
Constant	1.12 (.85, 1.40)	< .001	.50 (.24, .76)	< .001
Model statistics	$\chi^2(3) = 4.94$, $p = .176$<br>CD = .165<br>RMSEA = .012, 95% CI[< .001, .030], $p = 1.00$<br>CFI = .998<br>TLI = .975<br>AIC = 153,322.15<br>BIC = 154,928.20			

Note: CI = confidence intervals (lower limit, upper limit); LGBT = lesbian, gay, bisexual, transgender; -- = no path; CD = coefficient of determination; RMSEA = root mean square error of approximation; CFI = comparative fit index; TLI = Tucker-Lewis index; AIC = Akaike information criterion; BIC = Bayesian information criterion.

Supplementary Table 5. Study 1 Pulse nightclub massacre structural equation model standardized LGBT x Hispanic identity interaction indirect effects on acute stress through media exposure. $N = 4{,}675$

Variable	β (95% CI)	*p*
Age	-.01 (-.02, .01)	.209
Gender (comparison = male)		
Female	.001 (-.01, .01)	.866
Education (comparison = less than high school)		
High school	-.03 (-.06, -.001)	.042
Some college	-.05 (-.08, -.02)	.002
Bachelor's degree or higher	-.06 (-.10, -.03)	.001
Household income	-.01 (-.03, .001)	.066
Employment status (comparison = not working)		
Working	-.004 (-.02, .01)	.496
Marital status (comparison = married / cohabitating)		
Widowed / Divorced / Separated	-.003 (-.02, .01)	.704
Never married / Single	.02 (.01, .04)	< .001
Strength of political party affiliation (higher = stronger Democrat)	.02 (.01, .03)	.001
Prior self-reported mental health diagnoses (comparison = neither anxiety nor depression)		
Anxiety or depression	.01 (-.001, .02)	.061
Anxiety and depression	.004 (-.01, .02)	.508
Direct exposure to Pulse nightclub massacre (comparison = no)		
Yes	.05 (.04, .07)	< .001
LGBT identity (comparison = non-LGBT)		
LGBT	.01 (-.002, .03)	.083
Hispanic identity (comparison = non-Hispanic)		
Hispanic	.01 (-.0003, .03)	.055
LGBT x Hispanic identities	.01 (-.003, .03)	.124

Note: CI = confidence intervals (lower limit, upper limit); LGBT = lesbian, gay, bisexual, transgender.

Supplementary Table 6. Study 2 Ford-Kavanaugh hearings sample descriptive statistics. Weighted $N = 4{,}894$

Variable	Weighted *n* (%)	Weighted mean (*SD*)	95% CI	Range
Age		47.11 (17.15)	46.37, 47.85	18, 93
Ethnicity				
Black, non-Hispanic	535 (10.92)			
Hispanic	582 (11.89)			
Other, non-Hispanic	392 (8.02)			
Asian, non-Hispanic	165 (3.37)			
Other, non-Hispanic	77 (1.58)			
2+ races, non-Hispanic	150 (3.07)			
White, non-Hispanic	3,385 (69.17)			
Education				
Less than high school	445 (9.09)			
High school	1,406 (28.73)			
Some college	1,508 (30.80)			
Bachelor's degree or higher	1,535 (31.37)			
Household income		3.24 (1.93)	3.16, 3.31	1, 8
Less than $24,999	896 (18.32)			
$25,000 to $49,999	1,240 (25.35)			
$50,000 to $74,999	1,028 (21.00)			
$75,000 to $99,999	645 (13.17)			
$100,000 to $124,999	394 (8.05)			
$125,000 to $149,999	264 (5.39)			
$150,000 to $174,999	171 (3.48)			
$175,000 or more	257 (5.25)			
Employment status				
Not working (temp layoff, retired, disabled, other)	1,888 (38.58)			
Working (self-employed, as a paid employee)	3,006 (61.42)			
Marital status				
Married / Cohabitating	2,884 (58.94)			
Widowed / Divorced / Separated	892 (18.23)			
Never married / Single	1,117 (22.83)			
U.S. Census Bureau geographic region				

Northeast	886 (18.10)			
Midwest	1,079 (22.04)			
South	1,808 (36.95)			
West	1,121 (22.91)			
Interpersonal violence victim identity				
No	2,529 (52.39)			
Yes to any of the following:	2,298 (47.61)			
Sexual assault	1,491 (30.66)			
Rape	832 (17.20)			
Intimate partner violence	1,553 (31.96)			
Gender				
Male	2,351 (48.04)			
Female	2,543 (51.96)			
Strength of political party affiliation (higher = stronger Democrat)		4.16 (1.94)	4.08, 4.24	1, 7
Interpersonal violence victim X gender				
No, male	1,550 (32.12)			
No, female	979 (20.28)			
Yes, male	774 (16.02)			
Yes, female	1,525 (31.58)			
Self-reported daily hours of Ford-Kavanaugh hearings media exposure		6.58 (5.77)	6.34, 6.83	0, 25+
Self-reported Ford-Kavanaugh hearings-related acute stress		2.39 (3.74)	2.23, 2.55	0, 20

Note: *SD* = standard deviation; CI = confidence intervals (lower limit, upper limit); Range = minimum, maximum; Numbers may vary due to missing data.

Supplementary Table 7. Study 2 Ford-Kavanaugh hearings structural equation model standardized direct main effects. Weighted *N* = 4,894

	Path to media exposure		Path to acute stress	
Variable	β (95% CI)	*p*	β (95% CI)	*p*
Age	.01 (-.06, .07)	.851	-.15 (-.20, -.10)	< .001
Ethnicity (comparison = White, non-Hispanic)				
Black, non-Hispanic	.08 (.02, .14)	.008	.05 (.01, .10)	.011
Hispanic	.03 (-.02, .08)	.249	.03 (-.02, .09)	.188
Other, non-Hispanic	.01 (-.03, .05)	.774	.05 (.01, .10)	.023
Education (comparison = less than high school)				
High school	-.02 (-.14, .09)	.698	-.15 (-.27, -.04)	.009
Some college	.003 (-.11, .11)	.955	-.16 (-.27, -.04)	.007
Bachelor's degree or higher	.06 (-.06, .17)	.334	-.16 (-.27, -.05)	.006
Household income	.04 (-.01, .08)	.113	-.08 (-.12, -.04)	< .001
Employment status (comparison = not working)				
Working	-.06 (-.11, -.01)	.029	-.06 (-.11, -.01)	.010
Marital status (comparison = married / cohabitating)				
Widowed / Divorced / Separated	.01 (-.03, .04)	.764	-.03 (-.06, .01)	.110
Never married / Single	-.003 (-.06, .06)	.928	-.01 (-.06, .04)	.641
U.S. Census-designated geographic region (comparison = Northeast)				
Midwest	--	--	.01 (-.04, .06)	.770
South	--	--	-.01 (-.06, .04)	.678
West	--	--	.0003 (-.05, .05)	.990
Interpersonal violence victim (comparison = no)				
Yes	.13 (.09, .18)	< .001	.23 (.19, .27)	< .001
Gender (comparison = male)				
Female	-.08 (-.13, -.04)	< .001	.04 (.01, .08)	.024
Strength of political party affiliation (higher = stronger Democrat)	.10 (.05, .14)	< .001	.10 (.06, .14)	< .001
Self-reported daily hours of Ford-Kavanaugh hearings media exposure	--	--	.22 (.17, .27)	< .001
Constant	1.03 (.66, 1.39)	< .001	1.02 (.67, 1.36)	< .001
Model statistics	CD = .191			

Note: CI = confidence intervals (lower limit, upper limit); -- = no path; CD = coefficient of determination.

Supplementary Table 8. Study 2 Ford-Kavanaugh hearings structural equation model standardized indirect effects on acute stress through media exposure. Weighted *N* = 4,894

Variable	β (95% CI)	*p*
Age	.001 (-.01, .02)	.851
Ethnicity (comparison = White, non-Hispanic)		
Black, non-Hispanic	.02 (.003, .03)	.016
Hispanic	.01 (-.004, .02)	.255
Other, non-Hispanic	.001 (-.01, .01)	.774
Education (comparison = less than high school)		
High school	-.01 (-.03, .02)	.700
Some college	.001 (-.02, .02)	.955
Bachelor's degree or higher	.01 (-.01, .04)	.330
Household income	.01 (-.002, .02)	.122
Employment status (comparison = not working)		
Working	-.01 (-.02, -.001)	.031
Marital status (comparison = married / cohabitating)		
Widowed / Divorced / Separated	.001 (-.01, .01)	.764
Never married / Single	-.001 (-.01, .01)	.928
Interpersonal violence victim (comparison = no)		
Yes	.03 (.02, .04)	< .001
Gender (comparison = male)		
Female	-.02 (-.03, -.01)	.001
Strength of political party affiliation (higher = stronger Democrat)	.02 (.01, .03)	< .001

Note: CI = confidence intervals (lower limit, upper limit).

Supplementary Table 9. Study 2 Ford-Kavanaugh hearings structural equation model standardized interpersonal violence victim, gender, and political identity interactions direct main effects. Weighted $N = 4,894$

	Path to media exposure		Path to acute stress	
Variable	β (95% CI)	*p*	β (95% CI)	*p*
Age	.01 (-.06, .07)	.826	-.15 (-.20, -.10)	< .001
Ethnicity (comparison = White, non-Hispanic)				
Black, non-Hispanic	.08 (.02, .14)	.008	.05 (.01, .09)	.021
Hispanic	.03 (-.02, .08)	.250	.03 (-.02, .09)	.191
Other, non-Hispanic	.01 (-.03, .04)	.798	.06 (.01, .10)	.019
Education (comparison = less than high school)				
High school	-.02 (-.14, .09)	.709	-.16 (-.27, -.04)	.006
Some college	.004 (-.11, .12)	.930	-.16 (-.28, -.05)	.004
Bachelor's degree or higher	.06 (-.06, .17)	.323	-.17 (-.28, -.05)	.004
Household income	.04 (-.01, .08)	.132	-.07 (-.11, -.03)	< .001
Employment status (comparison = not working)				
Working	-.06 (-.11, -.01)	.029	-.06 (-.11, -.02)	.009
Marital status (comparison = married / cohabitating)				
Widowed / Divorced / Separated	.004 (-.03, .04)	.803	-.02 (-.06, .01)	.163
Never married / Single	-.004 (-.06, .06)	.905	-.01 (-.06, .04)	.708
U.S. Census-designated geographic region (comparison = Northeast)				
Midwest	--	--	.01 (-.05, .06)	.841
South	--	--	-.01 (-.06, .04)	.726
West	--	--	.0003 (-.05, .05)	.991
Interpersonal violence victim (comparison = no)				
Yes	.15 (.09, .21)	< .001	.21 (.15, .26)	< .001
Gender (comparison = male)				
Female	-.07 (-.13, -.01)	.033	.03 (-.02, .07)	.247
Strength of political party affiliation (higher = stronger Democrat)	.11 (.05, .17)	< .001	.01 (-.05, .07)	.652
Interpersonal violence victim x female	-.03 (-.11, .05)	.434	.04 (-.03, .11)	.290
Interpersonal violence victim x political party affiliation	-.04 (-.10, .02)	.175	.10 (.05, .16)	< .001
Female x political party affiliation	.01 (-.05, .08)	.651	.02 (-.04, .08)	.454
Self-reported daily hours of Ford-Kavanaugh hearings media exposure	--	--	.22 (.17, .27)	< .001
Constant	1.02 (.65, 1.38)	< .001	1.04 (.70, 1.37)	< .001
Model statistics	CD = .199			

Note: CI = confidence intervals (lower limit, upper limit); -- = no path; CD = coefficient of determination.

Supplementary Table 10. Study 2 Ford-Kavanaugh hearings structural equation model standardized interpersonal violence victim, gender, and political identity interaction indirect effects on acute stress through media exposure. Weighted *N* = 4,894

Variable	β (95% CI)	*p*
Age	.002 (-.01, .02)	.826
Ethnicity (comparison = White, non-Hispanic)		
Black, non-Hispanic	.02 (.003, .03)	.016
Hispanic	.01 (-.004, .02)	.256
Other, non-Hispanic	.001 (-.01, .01)	.798
Education (comparison = less than high school)		
High school	-.004 (-.03, .02)	.710
Some college	.001 (-.02, .03)	.930
Bachelor's degree or higher	.01 (-.01, .04)	.320
Household income	.01 (-.003, .02)	.141
Employment status (comparison = not working)		
Working	-.01 (-.02, -.001)	.031
Marital status (comparison = married / cohabitating)		
Widowed / Divorced / Separated	.001 (-.01, .01)	.803
Never married / Single	-.001 (-.01, .01)	.904
Interpersonal violence victim (comparison = no)		
Yes	.03 (.02, .05)	< .001
Gender (comparison = male)		
Female	-.01 (-.03, -.0004)	.043
Strength of political party affiliation (higher = stronger Democrat)	.02 (.01, .04)	< .001
Interpersonal violence victim x female	-.01 (-.02, .01)	.432
Interpersonal violence victim x political party affiliation	-.01 (-.02, .004)	.176
Female x political party affiliation	.003 (-.01, .02)	.648

Note: CI = confidence intervals (lower limit, upper limit).
